# PHY domain governs structural and photochemical fidelity in the far-red-absorbing state of phytochromes

**DOI:** 10.3389/fmolb.2026.1753512

**Published:** 2026-02-02

**Authors:** Tobias Fischer, Lisa Köhler, Florian Trunk, Qian-Zhao Xu, Kai-Hong Zhao, Valentin Rohr, Jörg Matysik, Wolfgang Gärtner, Josef Wachtveitl, Chen Song, Chavdar Slavov

**Affiliations:** 1 Institute of Physical and Theoretical Chemistry, Goethe University, Frankfurt, Germany; 2 Institute for Analytical Chemistry, University of Leipzig, Leipzig, Germany; 3 Key State Laboratory of Agriculture Microbiology, Huazhong Agriculture University Wuhan, Wuhan, China; 4 Department of Chemistry, University of South Florida, Tampa, FL, United States

**Keywords:** chromophore heterogeneity, photoconversion dynamics, phytochrome photoreceptors, solid-state NMR spectroscopy, ultrafast spectroscopy

## Abstract

**Background:**

Despite its central role in signaling, the influence of protein architecture on phytochrome structure and reactivity remains poorly understood. Here, we test how removal of the PHY domain reshapes the far-red–absorbing P_fr_ energy landscape and photochemical branching in the knotless phytochrome All2699g1g2.

**Methods:**

We combined femtosecond transient absorption (TA) spectroscopy with solid-state NMR to compare P_fr_ chromophore conformations and photodynamics in a GAF1–PHY construct versus the isolated GAF1 domain. Model-independent lifetime density maps and kinetic modeling were used to resolve the relaxation pathways and the population-weighted photoproduct yields.

**Results:**

GAF1–PHY displays a single chromophore conformation with homogeneous photodynamics and a photoconversion quantum yield of 16%. In contrast, GAF1-only exhibits three ground-state subpopulations (NMR) and heterogeneous photodynamics (TA), with kinetically distinct excited-state behaviors and markedly different branching toward Lumi-F photoproduct formation. One subpopulation accounts for ∼95% of photoproduct formation, whereas the other two relax predominantly through nonproductive recovery, yielding an overall photoconversion quantum yield of ∼10%. The productive branch shows a strongly red-shifted stimulated emission consistent with transient deprotonation at ring C or D, and the GAF1-only photoproduct exhibits CBCR-like electronic rearrangements relative to the canonical PHY-stabilized pattern.

**Conclusion:**

The PHY domain acts as a structural gatekeeper that suppresses intrinsic chromophore heterogeneity and directs P_fr_ excited-state evolution into a defined, productive photoconversion pathway. These findings provide a mechanistic foundation for domain-level control of photoreceptor function and future engineering of light-responsive proteins and optogenetic tools.

## 1 Introduction

**FIGURE 1:**
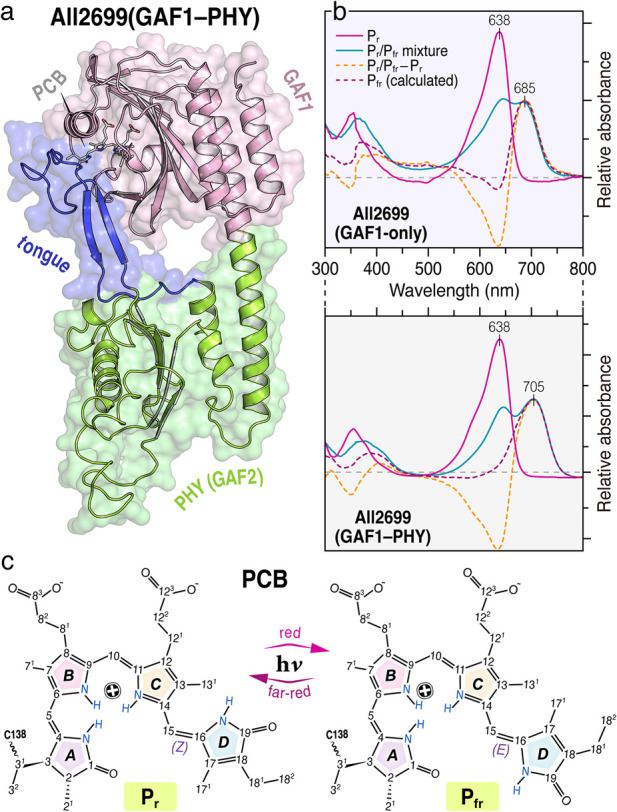
**(a)** Homology model of the GAF1–PHY construct of All2699 in the P_r_ dark state (Xu Q.-Z. et al., 2019). The PCB chromophore (*grey*) embedded in the GAF1 domain (*pink*) is partially sealed from solvent access by a tongue-like region (blue) protruding from the PHY domain (green). **(b)** UV/vis spectra of the two constructs. See SI for experimental conditions. **(c)** PCB in its protein-bound P_r_ *ZZZssa* and P_fr_ *ZZEssa* geometries.

Phytochromes are bilin-binding photoreceptors that regulate essential light-dependent processes such as photomorphogenesis, phototaxis, photosynthesis, and photoprotection in plants, cyanobacteria, bacteria, algae, and fungi (Rockwell and Lagarias, 2006; Rockwell et al., 2006; Möglich et al., 2010; Anders and Essen, 2015). Their activity is driven by a reversible *Z/E* isomerization of a covalently attached linear tetrapyrrole chromophore. The specific chromophore—phytochromobilin (PΦB), biliverdin (BV), or phycocyanobilin (PCB)—depends on the organismal lineage. Based on their domain architecture, phytochromes are classified into canonical (Group I), knotless (Group II) and cyanobacteriochromes (CBCRs, Group III). Group I phytochromes, including plant (e.g. PhyA, PhyB), some cyanobacterial (e.g. *Syn*Cph1, CphA), fungal, and bacteriophytochromes (e.g. *Ag*p1, *Dr*BphP), feature a photosensory core module (PCM) composed of PAS, GAF, and PHY domains (Rockwell and Lagarias, 2006; Rockwell et al., 2006; Möglich et al., 2010; Anders and Essen, 2015). The chromophore is embedded within the GAF domain; the PAS domain forms a distinctive figure-of-eight knot with the GAF domain; and the PHY domain regulates chromophore shielding and conformational dynamics via a flexible, tongue-like structure (Montgomery and Lagarias, 2002; Essen et al., 2008; Anders and Essen, 2015; Rockwell and Lagarias, 2017). In contrast, Group II phytochromes lack the PAS domain and are thus referred to as knotless or Cph2-like phytochromes (Montgomery and Lagarias, 2002; Rockwell and Lagarias, 2006; Anders et al., 2011; Anders and Essen, 2015). CBCRs (Group III) are composed of tandem GAF domains, one or more of which can bind a chromophore. CBCRs exhibit remarkable spectral diversity spanning the ultraviolet to near-infrared range, whereas canonical and knotless phytochromes convert almost exclusively between red-absorbing (P_r_) and far-red-absorbing (P_fr_) states (Chai et al., 1987; Lamparter et al., 1997; 2002; Hirose et al., 2008; Anders et al., 2011; Rockwell et al., 2011; 2012; 2014; 2016; Duanmu et al., 2014; Fushimi et al., 2017; Bandara et al., 2021; Niu et al., 2024). Notably, some isolated GAF domains of Group II and III phytochromes retain photoconversion capability (Ikeuchi and Ishizuka, 2008). Despite the extensive structural and spectroscopic characterization, the relationship between ground-state structural heterogeneity (von Stetten et al., 2008; Song et al., 2011a; 2015; Gourinchas et al., 2017; Xu Q.-Z. et al., 2019; Xu X. et al., 2020) and excited-state photodynamics (Kim et al., 2014b; Slavov et al., 2015c; Buhrke et al., 2020; Kirpich et al., 2021; Tachibana et al., 2021) in phytochromes remains poorly understood. Several Group I phytochromes, once thought to exhibit a homogeneous P_fr_ state, have been shown—using solid-state NMR, resonance Raman, and CD spectroscopy—to adopt multiple substates with distinct chromophore conformations, particularly in the *C-D* and *A-B* methine bridge regions (Song et al., 2011a; Spillane et al., 2012; Salewski et al., 2013; Rockwell et al., 2014; Velazquez Escobar et al., 2015; Stensitzki et al., 2017). These findings raise important questions about whether such ground-state heterogeneity translates into functionally distinct photochemical pathways. Despite structural heterogeneity in various phytochromes, ultrafast studies of the P_fr_-to-P_r_ photoconversion often reveal surprisingly homogeneous excited-state dynamics, suggesting that energetic barriers between ground-state substates are easily overcome upon excitation. In PhyA, for instance, heterogeneity involving a ∼10° shift in the *C-D* dihedral angle was proposed to create a slower or nonreactive decay channel in the P_fr_ excited state (Velazquez Escobar et al., 2015). However, no corresponding kinetic bifurcation has been detected experimentally (Bischoff et al., 2001; Müller et al., 2008). In the case of Cph1, multiphasic excited-state dynamics have been attributed to ground-state heterogeneity in the form of coexisting reactive and non-reactive chromophore conformations (Kim et al., 2014a; Stensitzki et al., 2017). Conversely, a study on bacteriophytochromes (*Pa*BphP and *Rp*BphP2) reported that, despite pronounced ground-state heterogeneity, the excited-state dynamics progresses homogeneously (Wang et al., 2016). In other phytochromes, such as *Ag*p1 and *Pa*BphP, multiphasic behavior has instead been linked to branching pathways on the excited-state potential energy surface (Schumann et al., 2008; Wang et al., 2019). Recent studies on knotless phytochromes have added further complexity. Our previous work on the PCM of *Syn*Cph2 revealed homogeneous P_fr_ photodynamics (Fischer et al., 2021). However, solid-state NMR studies or the analogous All2699g1g2 (Figure 1) indicated that removal of the PHY domain induces significant structural heterogeneity of the P_r_ state (Xu Q.-Z. et al., 2019). Despite that, the primary photodynamics of P_r_ proceeds homogeneously in both the GAF1-only All2699g1 and the GAF1–PHY construct All2699g1g2 (Fischer et al., 2020; Slavov et al., 2020). This raises the compelling question: *Does removal of the PHY domain induce structural heterogeneity in the P*_*fr*_ *state, and if so, how does it impact the ultrafast P*_*fr*_ *photodynamics?* To address this, we compare the P_fr_-state dynamics of the full-length All2699g1g2 knotless phytochrome (hereafter referred to as GAF1–PHY) and its single-domain counterpart All2699g1 (GAF1-only) using ultrafast UV/vis spectroscopy. By integrating these kinetic measurements with structural data from solid-state NMR, we elucidate how domain architecture modulates ground-state heterogeneity and whether such heterogeneity governs the primary photoreaction on ultrafast timescales. This combined structural-dynamic approach provides new insight into the functional role of the PHY domain and addresses a long-standing ambiguity in phytochrome signaling.

## 2 Materials and methods

### 2.1 Sample preparation

Preparation of the isolated GAF1-only and bidomain GAF1–PHY constructs of All2699 protein are described elsewhere (Xu Q.-Z. et al., 2019; Slavov et al., 2020). For solid-state NMR spectroscopy, two All2699 holoproteins *in vitro* assembled with uniformly ^13^C- and ^15^N-labeled PCB [*u*-[^13^C,^15^N]-PCB-All2699(GAF1) and *u*-[^13^C,^15^N]-PCB-All2699(GAF1–PHY)] as lyophilized powder were used (Xu Q.-Z. et al., 2019). No additional illumination was applied during the NMR acquisition. For ultrafast transient absorption spectroscopy, the *in vivo* assembled holoproteins were prepared in a final buffer containing 50 mM Tris, 150 mM NaCl, and 5% glycerol at pH 8.0.

### 2.2 NMR spectroscopy

All solid-state NMR experiments were acquired on a wide-bore Bruker AVANCE-III 400 MHz spectrometer equipped with a 4 mm double-resonance magic-angle spinning (MAS) probe (Rheinstetten, Germany). All spectra were externally referenced to the COO^−^ signal of solid _L_-Tyrosine·HCl at 172.1 ppm. The data was preprocessed with Bruker Topspin 4.0.1 and further analyzed with MestReNova 14.1.0 (Mestrelab Research, Santiago de Compostella, Spain).

### 2.3 Ultrafast transient absorption spectroscopy

The time-resolved transient absorption experiments were performed using a home-built pump-probe setup which was described previously elsewhere (Slavov et al., 2015a). In the set-up a Ti:sapphire amplifier system (Clark, MXR-CPA-iSeries) was used to generate the fundamental laser pulses (775 nm, 130 fs, 1 kHz) which are used to generate the pump and probe pulses. The pump pulses are generated using a home-built two stage NOPA (noncollinear optical parametric amplifier) with a prism compressor in between the two NOPA stages for pulse compression. For probing, white-light continuum pulses were generated by focusing the laser fundamental into a CaF_2_-crystal (5 mm). A time resolution of ∼100 fs for the experiment was estimated from the pump-probe cross correlation. The experiments were conducted under magic angle conditions (54.7° pump-probe polarization difference) with excitation pulses at 710 nm and 70 nJ/pulse and probe energy 10–20 pJ/nm or lower. Samples were placed in a 1 mm path-length fused-silica cuvette and continuously translated at high speed in two orthogonal directions within a plane perpendicular to the probe beam. A high-power 625 nm LED (Thorlabs, 1 W) provided continuous illumination to maintain the sample in the P_fr_ state and prevent photoproduct accumulation. The analysis of the transient absorption data was performed using OPTIMUS (www.optimus.optimusfit.org) (Slavov et al., 2015b).

## 3 Results and discussion

### 3.1 Homogeneous P_fr_ dynamics of the GAF1–PHY construct

**FIGURE 2:**
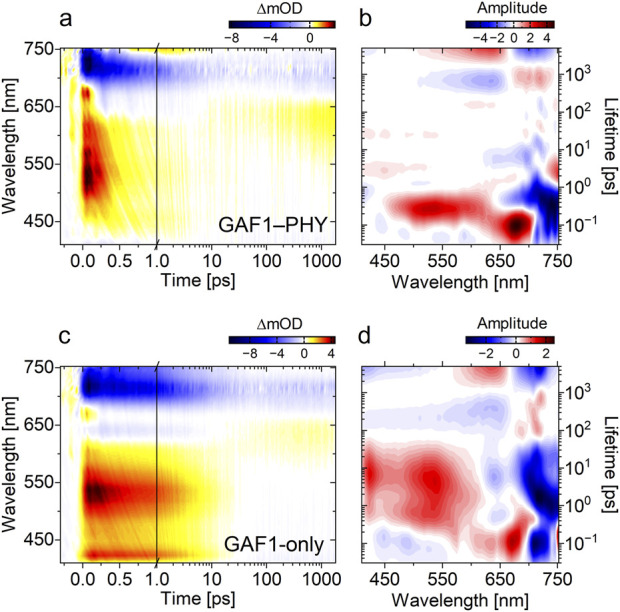
P_fr_ transient absorption data of GAF1–PHY **(a)** and GAF1-only **(c)** after 710 nm excitation. **(b,d)** Corresponding lifetime density maps (LDM) derived from the lifetime distribution analysis of the data in **(a,c)**, correspondingly. In the transient absorption data, positive amplitudes (red) reflect excited state absorption (ESA) and photoproduct absorption (PA), while negative amplitudes (blue) correspond to ground state bleach (GSB) or stimulated emission (SE). In the LDMs, negative amplitudes indicate either rise of ESA/PA or decay of GSB/SE, whereas positive amplitudes represent decay of ESA/PA or rise of GSB/SE.

In most Group I and II phytochromes, the ultrafast P_fr_ → P_r_ photodynamics occurs on a similar timescale and is significantly faster than the P_r_ → P_fr_ reaction. After rapid departure from the Franck-Condon region (∼100 fs), the excited state relaxes within ∼500–700 fs to form the primary photointermediate Lumi-F (Bischoff et al., 2001; Heyne et al., 2002; Müller et al., 2008; Schumann et al., 2008; Kim et al., 2014b; Yang et al., 2016; Stensitzki et al., 2017), which subsequently generates the blue-shifted Meta-F intermediate (Müller et al., 2008; Kim et al., 2014b; Stensitzki et al., 2017). The P_fr_ ultrafast data (see SI for experimental details) of the GAF1-PHY construct is dominated by four transient absorption signals (Figure 2a). The broad positive signal from 450–650 nm corresponds to excited state absorption (ESA), while the negative feature at 700–750 nm arises from P_fr_ ground state bleach (GSB) and stimulated emission (SE). After ∼0.5 ps, a new positive signal emerges at 740 nm (faint yellow feature, Figure 2a), assigned to the primary photointermediate. By 5 ps, this intermediate decays, giving rise to a second positive signal at 550–600 nm, which persists beyond the experimental time window. We analyzed the transient absorption data using lifetime density analysis to resolve the details of underlying kinetics (see SI and ref (Slavov et al., 2015b). At ∼100 fs, the combination of negative (700–750 nm) and positive (650–700 nm) amplitude lifetime distributions indicate the departure of the wavepacket from the Franck-Condon region (Figure 2b). This is followed by a spectrally broad positive distribution from 450 to 600 nm and a negative distribution above 650 nm, reflecting the 300-fs decay of ESA and GSB/SE. Concurrently, a strong negative distribution above 730 nm marks the emergence of the primary hot ground-state intermediate (GSI), Lumi-F. This intermediate undergoes a spectral blue shift with a lifetime of 5–10 ps, as indicated by the distribution at 650–710 nm and the positive distribution at 740 nm. The blue shift continues on longer timescales, as illustrated by the positive distribution at 675–740 nm and the negative distribution at 525–650 nm, resulting in a final set of lifetime distributions. These correspond to the long-lived GSI, Meta-F, whose lifetime exceeds the detection window of our experiment. The overall kinetic behavior is highly similar to our previous observations in *Syn*Cph2—a knotless phytochrome also composed of GAF and PHY domains—and appears predominantly homogeneous (Fischer et al., 2021).

### 3.2 Heterogenous P_fr_ dynamics of the GAF1-only construct

In the ultrafast dynamics of the GAF1-only construct, only three transient absorption features are observed, as the positive signal above 730 nm seen the GAF1–PHY data is not clearly detectable (Figure 2c). Notably, the decay of the ESA below 600 nm is markedly prolonged into the picosecond range and includes an additional, well-defined band at 425 nm that is absent in the GAF1–PHY data. The lifetime density analysis (Figure 2d) reveals a complex pattern of lifetime distributions for both ESA and GSB signals. While the ∼100 fs Franck-Condon relaxation is preserved, the relatively narrow lifetime distribution reflecting the decay of the ESA, and the GSB/SE signals in the GAF1–PHY data is replaced by an extended and very structured distribution spanning from ∼500 fs to ∼20 ps (compare Figures 2b,c). These positive- and negative distributions can be assigned to a multiphasic P_fr_ excited-state decay associated with elevated recovery of the ground state and reduced formation of the primary GSI. The extension of the strong negative distribution to longer wavelengths suggests some formation of the red-shifted primary GSI, similar to that observed in the GAF1–PHY data (Figure 2a) and in *Syn*Cph2 (Fischer et al., 2021). The later phases of the excited-state decay, represented by the positive distributions in the 2–20 ps lifetime range, coincide with the early blue spectral shift evolution of the primary photoproduct. This is evident by the dip in the lifetime distribution at 3–10 ps (>730 nm). This dip is the result of the overlap between the negative distribution (SE decay) and the positive distribution (photoproduct blue shift). This data suggests that the slowest components of excited-state decay persist beyond the early evolution of the primary photoproduct. As a result, it is difficult to determine their exact contribution to product formation. Nevertheless, both excited-state decay and photoproduct formation appear substantially stretched in time and heterogenous compared to the P_fr_ dynamics of the GAF1–PHY construct and *Syn*Cph2. A detailed kinetic model will require structural information to account for this heterogeneity.

### 3.3 Homogeneous vs. heterogenous P_fr_ kinetics of All2699

**FIGURE 3:**
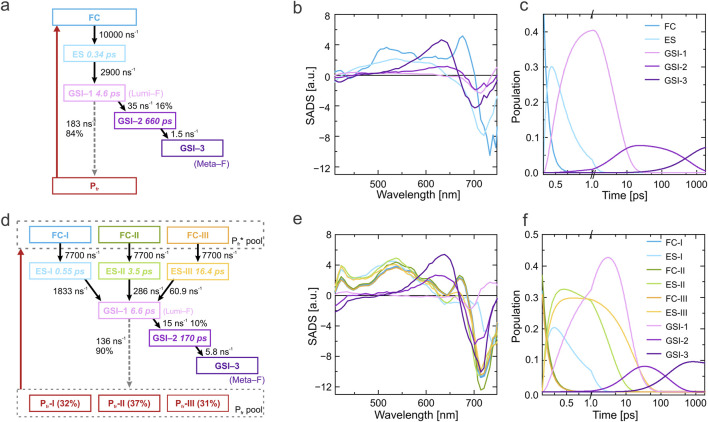
Kinetic modeling of the P_fr_ dynamics. **(a–c)** show the schematic representation of the kinetic model, species-associated difference spectra (SADS) and the corresponding populations for GAF1–PHY P_fr_. **(d–f)** show the schematic representation of the kinetic model, SADS and the corresponding populations for GAF1-only P_fr_. Given that the FC SADS are spectrally indistinguishable, and we find no evidence for appreciable differences in the absorption cross sections of the three subpopulations, it is reasonable to assume comparable excitation selectivity. Accordingly, the fitted excitation vector (0.32:0.37:0.31 for FC-I:FC-II:FC-III) can be taken as a practical estimate for the ground-state P_fr_-pool distribution. For more details, see Supplementary Figures S3–S5.

Guided by the qualitative insights from the LDMs (Figures 2b,d), we constructed kinetic models for the GAF1-only and GAF1–PHY P_fr_ states, enabling extraction of rate constants and spectral signatures of the underlying species (Figure 3).

The LDM of the GAF1–PHY P_fr_ dynamics revealed a single excited-state decay component, consistent with a homogenous chromophore population. This justified the construction of a global analysis model based on a single ground-state configuration (Fischer et al., 2021). To capture the ultrafast relaxation, the model included a Franck–Condon (FC) state, a short-lived relaxed excited state minimum, and three consecutive ground-state intermediates (GSIs) (Figure 3a). Constraining the model by the experimentally determined quantum yield of P_fr_ photoconversion (16%, see Supplementary Figure S1 and SI for details on quantum yield determination) reduced parameter space and yielded physically meaningful rate constants and species-associated difference spectra (SADS) (Figures 3a,b).

Strikingly, this homogenous model closely mirrors that of the related knotless phytochrome Cph2, with nearly identical excited-state lifetimes (0.34 ps vs. 0.33 ps) and similar intermediate spectral features (Fischer et al., 2021). Unlike Cph2, however, we could not resolve a distinct Lumi-F_hot_ state, likely due to the shorter lifetime of Lumi-F (4.6 ps). We assign GSI-1 to Lumi-F based on its red-shifted absorption (>730 nm) (Figure 3b). Lumi-F proceeds via a single intermediate (GSI-2, 640 nm) to reach Meta-F (GSI-3, 630 nm), suggesting a more direct ground-state evolution in GAF1–PHY.

In contrast, the GAF1-only construct exhibited strikingly heterogeneous P_fr_ ultrafast dynamics. The transient absorption data and the corresponding LDMs (Figures 2c,d) revealed multiple decay components with substantially different timescales, ranging from sub-ps to tens of picoseconds. These differences could not be reconciled with a single homogeneous population and instead pointed toward the presence of multiple distinct ground-state configurations, giving rise to spectro-kinetically distinguishable excited-state behaviors. To account for this, we constructed a kinetic model incorporating three parallel P_fr_ ground state populations (P_fr_-I, P_fr_-II, and P_fr_-III), each contributing nearly equally to the observed transient kinetics (Figure 3d). It is worth noting that the NMR analysis (*vide infra*) also independently revealed three subpopulations with an approximately 1:1:1 distribution. To ensure physically reasonable results and account for the presence of a shunt pathway, we limited the total photoproduct quantum yield to 10% (Trunk et al., 2025) (see SI for details). The Franck-Condon spectra (FC-I, FC-II, and FC-III) are essentially identical and the excited states (ES-I, ES-II, and ES-III) of the corresponding P_fr_ populations share similar ESA features but differ markedly in decay kinetics (0.55 ps, 3.5 ps, and 16.4 ps, respectively) and spectral bleach patterns (Figures 3d,e), suggesting structurally encoded relaxation differences. Consequently, we developed a model in which all ESs funnel into a shared primary intermediate (GSI-1, analogous to GAF1–PHY and Cph2 Lumi-F), which then either relaxes back to the initial P_fr_ pool or proceeds forward along a common pathway towards Meta-F (Figure 3d).

We then attempted to determine the photoconversion efficiencies of each subpopulation. For this purpose, we tested a fully heterogenous model with completely independent product formation pathways for each subpopulation (Supplementary Figure S5). While the model fits the data, it is severely overparametrized, which hinders the reliable recovery of some of the kinetic rates and the branching efficiencies. To address this limitation, we implemented a simplified model in which each ES either relaxes back to its original P_fr_ state or proceeds towards a shared GSI-1 (Supplementary Figure S4). The purpose of this model is not to provide mechanistic insight but solely to extract the branching efficiencies for each subpopulation while avoiding harsh increase in model complexity and the resulting overparameterization. Remarkably, P_fr_-I, accounting for just 32% of the initial P_fr_ population, produces ∼95% of the final photoproduct, whereas P_fr_-II contributes only ∼5% and P_fr_-III is virtually non-productive. The ∼30% branching toward photoproduct formation in P_fr_-I (Figure 3d) does not imply ‘enhanced’ or ‘penalized’ photochemistry overall, but rather reflects the intrinsic productivity of one subpopulation within a heterogeneous ensemble (overall population-weighted quantum yield of ∼10%). Interestingly, the decay dynamics of P_fr_-I resemble those of the GAF1–PHY construct, suggesting that this subpopulation may have structural features stabilized by the PHY domain. Notably, the productive population shows a strongly red-shifted SE, consistent with a potential transient deprotonation of ring *C* or *D* (Yang et al., 2022), although explaining why P_fr_-I is comparatively more productive will require additional structural and theoretical data beyond the scope of this work. Together, these results indicate that, in the absence of the PHY domain, the chromophore binding pocket permits conformational diversity, leading to heterogeneous and overall inefficient photoconversion, whereas the GAF1–PHY architecture constrains the intrinsic bilin flexibility to enforce structural and kinetic uniformity and ensure a defined, well-regulated photosensory output.

### 3.4 Conformational consistency vs. structural heterogeneity

**FIGURE 4:**
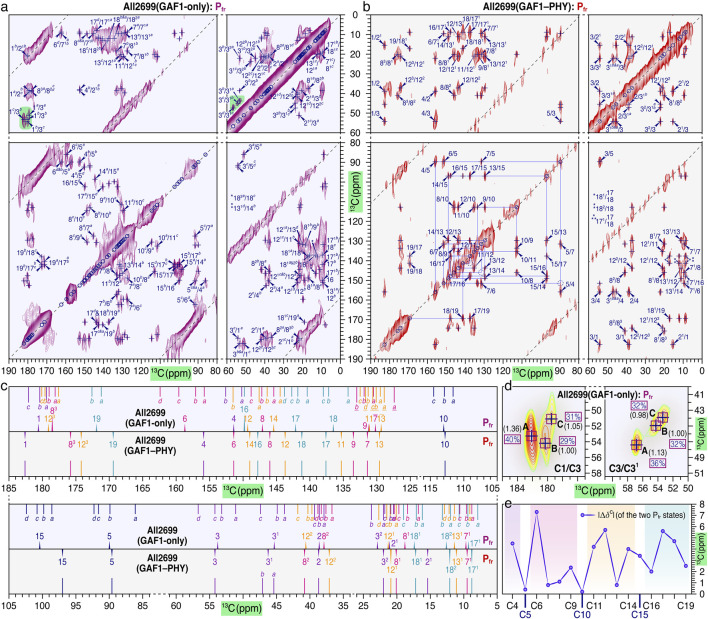
Structural heterogeneity vs. homogeneity at bilin carbons of P_fr_ in All2699. 2D ^13^C–^13^C DARR spectra of **(a)** GAF1-only and **(b)** bidomain GAF1–PHY lyophilizates. Calculated spectra of the pure P_fr_ states were obtained by subtracting the corresponding P_r_ spectra at 100% occupancy (Xu Q.-Z. et al., 2019) from those of the P_r_/P_fr_ photoequilibrium mixtures with a weighting constant of ∼0.45 (*see* SI NMR). For clarity, only short- and medium-range carbon pairs of the PCB chromophore (see Figure 1C for numbering) are assigned with labels in blue. The complete ^13^C assignments of both constructs are given in Supplementary Figure S6. Observed ^13^C signal splittings are indicated by superscripts *a*, *b*, *c*, and so on from the high-to low-field side. The solid blue lines shown in panel **(b)** indicate sequence of short-range (directly-bonded) correlations for tracing ^13^C connectivities of the *π*-conjugated C4–C19 system of the bilin in the GAF1–PHY construct. **(c)** Multiple ^13^C resonances of a given bilin carbon and their mean chemical shifts are represented by solid lines and triangles, respectively, and are color-coded as shown in Figure 1C. **(d)** Enlarged views of the spectral regions of the C1/C3 and C3/C3^1^ correlations in the GAF1-only spectrum [shaded green, **(a)**]. The relative integrated intensities of the three different components (indicated as **A–C**) in each carbon pair are given in parentheses. The relative ratio obtained from the short-range C3/C3^1^ correlations has been used to quantitatively calculate the percentage of ground-state subpopulations of the GAF1-only P_fr_ (as indicated by blue numbers enclosed in magenta boxes). **(e)** Changes in the ^13^C chemical shift (Δ*δ* ^C^) upon inclusion of the PHY domain for the P_fr_ state. Δ*δ* ^C^ values for the bilin-conjugated C4–C19 system were calculated as *δ* ^C^_GAF1–PHY_ − *δ* ^C^_GAF1_.The semi-transparent colored regions highlight carbon atoms belonging to rings *A*–*D*, color-coded as purple, pink, orange, and blue, respectively.

To reveal P_fr_ structural features in the two All2699 constructs, we applied solid-state NMR spectroscopy using uniformly ^13^C- and ^15^N-labeled PCB chromophore (*u*-[^13^C,^15^N]-PCB). Two-dimensional (2D) homonuclear ^13^C–^13^C dipolar correlation (DARR) (Figures 4a,b, see SI for sample preparation and experimental details) spectra were recorded with an optimized 50 ms mixing time to enable detection of both nearest-neighbor and medium-range correlations (∼3.3–3.7 Å). Due to this specific labeling pattern, the DARR data exclusively probes the intramolecular structure and heterogeneity of the chromophore itself in different protein environments. The detection range and assignment strategy are based on earlier studies on these proteins in their corresponding P_r_ states as frozen solution (Xu Q.-Z. et al., 2019) and lyophilized powder (Kim et al., 2020).

#### 3.4.1 Homogeneous chromophore structure in the GAF1–PHY P_fr_ state

The DARR spectrum of GAF1–PHY construct in the P_fr_ state shows no evidence of structural heterogeneity, with a single set of *δ* ^c^ values for nearly all PCB carbons (32 out of 33) (Figure 4b). In addition to these uniform chemical shifts, the spectrum reveals all expected short-range correlations between strongly-coupled ^13^C spins of the *π*-conjugated C4–C19 system, along with multiple long-range contacts such as C3/C6, C10/C13 and C17^1^/C19 at ∼3.4–3.6 Å (Figure 4b). Together, these features indicate a structurally homogeneous chromophore environment on the NMR timescale and align well with the uniform excited-state behavior observed in ultrafast spectroscopy (Figures 2a,b). The extensive correlations suggest efficient spin diffusion and support the presence of a compact and well-ordered chromophore-binding pocket. This high degree of conformational consistency likely results from stabilizing interaction between the GAF domain and the conserved tongue element of the PHY domain (Figure 1a), an characteristic shared with canonical phytochromes (Anders et al., 2014; Xu Q.-Z. et al., 2019; Fischer et al., 2020; Xu Q.-Z. et al., 2020).

#### 3.4.2 Striking chromophore heterogeneity in the GAF1-only P_fr_ state

In contrast to the uniform chromophore structure in the GAF1–PHY construct, removal of the PHY domain results in a considerable increase in conformational variability of the P_fr_ state. The DARR spectrum of the GAF1-only construct reveals pronounced ^13^C resonance splittings (‘multiple signals’) across the PCB chromophore (Figure 4a), with 19 of 33 carbon positions showing three sets of chemical shift (*δ* ^c^) values (Figure 4c; Supplementary Table S1), indicating the presence of three conformational subpopulations. Additional splittings (e.g., C3, C5, C6, C8^1^, C8^2^, C12, C15, and C17) showing doubled or quadrupled resonances suggest characteristic structural and dynamic variability. Notably, the large ^13^C resonance separations for C5 and C6 (Δ = 6.2 and 6.6 ppm, respectively) at the *A*–*B* methine bridge point to a coexistence of *R* and *S* configurations at the tetrahedral C3^1^ atom, consistent with previous findings in the P_r_ state (Xu Q.-Z. et al., 2019). This is further supported by the large separations for ring *A* carbons C3 and C3^1^ (Figure 4c). Significant bilin flexibility of the *B*-ring propionate allows its carboxylate group (C8^3^) to point away from the bilin (‘stretched form’) or toward the *B*–*C* plane (‘bent form’). The unexpected detection of a long-range C8^3*b*^/C7 correlation (∼4.6 Å, inferred from the 6OZA P_r_ structure (Slavov et al., 2020)) can be associated with downward bending of the propionate towards the pyrrole rings, thus shortening the effective C···C distance into the DARR detection range (Figure 4a). The other C8^3^ subpopulations (*a*, *c*, and *d*) show no such DARR correlations, implicating the stretched forms, e.g., with C7^1^ and C7–C10 (Figure 4a).

Quantifying the relative abundance of the identified P_fr_ chromophore subpopulations is key to understanding how structural heterogeneity affects phytochrome photochemistry and function. Therefore, we analyzed the cross-peak intensities for three coupled pairs such as C1/C3, C3/C3^1^, and C12^3^/C10. For the strongly coupled C3/C3^1^ spin pair, the relative peak intensities are largely unaffected by conformational-dependent internuclear C···C distances and spin-diffusion and are thus governed mainly by local dynamics (Huster et al., 2002; Manolikas et al., 2008; Akbey et al., 2012; Xu Y. et al., 2019). This allows reliable quantification of the relative composition of the P_fr_ ground-state subpopulations from the integrated peak volumes: **A: B: C** = 1.13 : 1.00: 0.98, i.e., 36%: 32%: 32% (Figure 4d). Similar ratios from C1/C3 (40%: 31%: 29%, Figure 4d) and C12^3^/C10 (33%: 34%: 33%) further support this distribution, despite minor differences caused by dipolar truncation effects of these weak couplings by the presence of strong couplings in the system (Manolikas et al., 2008). The appearance of multiple ground-state chromophore conformations upon PHY domain removal underscores its critical role in restricting conformational freedom and preserving a well-defined P_fr_ structure. Moreover, the NMR-derived population ratios establish a direct structural basis for the multiphasic excited-state dynamics of the GAF1-only construct, firmly linking them to underlying ground-state heterogeneity.

#### 3.4.3 The PHY domain influences the electronic structure of the chromophore

Beyond structural stabilization, the PHY domain also affects the chromophore’s electronic structure. Significant chemical shift differences (in the vicinity of the C5-methinerepresented by the unsigned measure for the magnitude, |Δ*δ* ^C^|) between P_fr_ states of the two constructs are observed at rings *C* and *D* and in the vicinity of the C5-methine bridge (Figure 4e). Pyrrolic ^13^C shifts are known to reflect chromophore conjugation and geometry (Xu Y. et al., 2019), indicating that the PHY domain alters both the conformation and the electronic structure of the chromophore, particularly the outer rings (e.g., the twist angles referring to the *B–C* plane). For example, the C17/C13^1^ correlation, a marker of *D*-ring twist in the 15*E* state (Xu Q.-Z. et al., 2019), appears solely in the GAF1-only spectrum (Figure 4a). This suggests greater *D*-ring twisting in the GAF1-only construct, bringing the C17 carbon further away from the C13^1^ partner on the *C*-ring. This *D*-ring motion, modifying the coupling of the conjugated C4–C19 system, likely contributes to the 20 nm blue shift in the P_fr_ absorption maximum in the absence of the PHY domain (Figure 1b). This agrees with previous mutational studies demonstrating that the PHY tongue region plays a critical role in tuning P_fr_ absorption (Anders et al., 2014; Xu Y. et al., 2019; Xu Q.-Z. et al., 2020).

#### 3.4.4 Divergent conjugation rearrangement in GAF1-only vs. GAF1–PHY: CBCR-like vs. canonical phytochrome behavior

**FIGURE 5:**
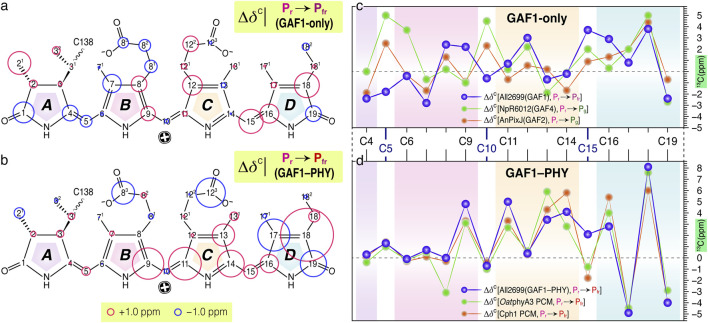
State-related Δ*δ* ^C^ changes of the PCB chromophore ^13^C resonances in All2699. The Δ*δ* ^C^ changes associated with P_r_ → P_fr_ photoconversion in **(a)** GAF1-only and **(b)** bidomain GAF1–PHY constructs are represented as red and blue circles for down- and upfield shifts (for the carbon atoms in the P_fr_ state), respectively. Comparisons of the corresponding Δ*δ* ^C^ changes in the conjugated C4–C19 system for **(c)** All2699GAF1 with the red/green CBCRs *Np*R6012GAF4 (Rockwell et al., 2015) and *An*PixJGAF2 (Altmayer et al., 2022) as well as for **(d)** All2699GAF1–PHY and the canonical *Oat*phyA3 (Song et al., 2018) and Cph1 (Rohmer et al., 2008) phytochromes. All displayed Δ*δ* ^C^ changes were calculated as *δ* ^C^_15*E*_ − *δ* ^C^_15*Z.*_

Based on the contrasting P_fr_ ground-state structures revealed by solid-state NMR, we analyzed the ^13^C chemical shift changes (Δ*δ* ^C^) associated with the C15-*Z*/*E* isomerization for the two All2699 constructs (Figures 5a,b). In the GAF1-only construct, Δ*δ* ^C^ values across most of the conjugated C4–C19 system remain modest (−2.8 to 2.4 ppm for 13 of 16 carbons) with the largest collective downfield shift of >2.9 ppm at C15, C16, and C18 (Figure 5a). These state-related changes, together with a smaller downfield shift at C17 (0.8 ppm), indicate a localized decrease of electron density at the C15-methine bridge and ring *D*. Strikingly, the Δ*δ* ^C^ pattern (and sign) over the C11–C19 segment—spanning rings *C* and *D* and the C15 bridge—closely mirrors that of the red/green CBCRs *Np*R6012GAF4 (Rockwell et al., 2015) and *An*PixJGAF2 (Altmayer et al., 2022) (Figure 5c), even though those proteins produce a green-absorbing P_g_ state. The resemblance suggests a shared photochemical mechanism related to similar rearrangement of the conjugated system and configuration of the formal C14, C15-single bond of the chromophore in the compared P_fr_ and P_g_ states. However, no such consistency is observed for the C4–C6 and C9–C10 regions of the conjugated system (Figure 5c). The large Δ*δ* ^C^ discrepancies occurring at the C5 (6.8 ppm) and C10 (5.1 ppm, minor P_r_-to-P_fr_ shift in All2699GAF1, but 4.5 ppm in *Np*R6102GAF4) methine bridges indicate different *A*-ring rotations and *B*/*C*-ring dynamics.

These results indicate that while the GAF1-only P_fr_ undergoes CBCR-like rearrangement around rings *C* and *D*, it lacks the additional structural motions near C10 (e.g., small torsion around C10–C11 bond and/or in-plane C10-methine bridge stretching) that are required for the formation of hypsochromic P_g_ states as in CBCRs. Thus, it adopts a CBCR-like electronic response without a corresponding spectral outcome. In stark contrast, the GAF1–PHY construct exhibits a Δ*δ* ^C^ pattern characteristic of canonical phytochromes. Upon photoconversion to the P_fr_ state, the major chemical shifts are largely localized at rings *C* and *D*, with minimal perturbation of rings *A* and *B* (Figure 5b; Supplementary Table S2). All four ^13^C resonances of ring *C* (C11–C14) shift downfield relative to P_r_, indicating a net loss of electron density and suggesting electron redistribution from ring *C* to other rings. An alternating Δ*δ* ^C^ pattern across ring *D* (Figure 5b) marks the extension of conjugation through the C15-methine bridge. This pattern closely matches that of canonical phytochromes such as Cph1 (Rohmer et al., 2008) and *Oat*phyA3 (Song et al., 2018) in both sign and magnitude (Figure 5d) and is an established hallmark of canonical P_r_-to-P_fr_ transitions. These distinct Δ*δ* ^C^ patterns observed between GAF1-only and GAF1–PHY constructs correlate with changes in the chromophore’s electronic structure and the pronounced bathochromic shift in the P_fr_ absorption maximum (Δ = 20 nm, Figure 1b). This shift, along with the conformational homogeneity consistency and narrowed linewidths in the GAF1–PHY construct (Supplementary Table S1), underscores the stabilizing and regulatory role of the PHY domain. Its conserved tongue, protruding towards the GAF1 domain (Xu Q.-Z. et al., 2019), enforces structural order and modulates electronic redistribution across the bilin, coordinating the physical and photochemical aspects of phytochrome signaling.

Our findings show that GAF1–PHY follows the canonical phytochrome ‘soft-to-hard’ mesoscopic phase transition upon photoconversion (Song et al., 2011b; 2018). Whereas removal of the PHY domain leads to increased local dynamics and a CBCR-like electronic rearrangement localized around rings *C* and *D*. The results emphasize the critical role of the PHY domain—and particularly its tongue element—in defining the chromophore’s rigidity, electronic structure, spectral properties, and photoconversion behavior.

## 4 Conclusion

Our combined ultrafast spectroscopy and solid-state NMR analysis reveals the profound impact of the PHY domain on the structure and the photochemical reactivity of the P_fr_ state. The GAF1–PHY construct exhibits a conformationally uniform chromophore ground state and a single photochemical pathway culminating in Meta-F formation. In contrast, removal of the PHY domain in GAF1-only induces striking ground-state heterogeneity, with at least three distinct chromophore conformers. These conformers give rise to spectroscopically resolvable excited-state trajectories with divergent lifetimes and photoproduct yields. Importantly, only one subpopulation accounts for nearly all productive photoconversion, while the others decay mostly through unproductive channels. The NMR data establishes that this heterogeneity arises from variations in methine bridge geometry (*R/S*-stereochemistry at the C3^1^ atom), propionate orientation, and the electronic coupling across the conjugated system. ^13^C chemical shift differences between the constructs reveal that the PHY domain imposes conformational constraint and promotes delocalization of electronic density over rings *C* and *D*—features that correlate with a red-shifted absorption maximum. The GAF1-only construct instead displays CBCR-like conjugation shifts and increased *D*-ring twist, underscoring the functional divergence caused by domain architecture. This is also reflected by the different quantum efficiency of photoisomerization found for the productive populations of GAF1-only and GAF1–PHY. Together, our findings define the PHY domain as a structural and electronic gatekeeper that limits conformational freedom, tunes chromophore conjugation and energetics, and directs excited-state evolution. This work not only clarifies the mechanistic role of the PHY domain in P_fr_ but also provides design insights for engineering light-responsive proteins with tailored structural and photochemical properties.

## Data Availability

The raw data supporting the conclusions of this article will be made available by the authors, without undue reservation.
