# Supplementary material for: PHY domain governs structural and photochemical fidelity in the far-red-absorbing state of phytochromes
Source: Front Mol Biosci. 2026 Feb 2;13:1753512. doi: 10.3389/fmolb.2026.1753512 (PMC12907550; doi:10.3389/fmolb.2026.1753512)
Supplement: Supplementary file 1 [file DataSheet1.pdf]

Supplementary Material

PHY Domain Governs Structural and Photochemical Fidelity in the Far-Red-Absorbing State of Phytochromes

Tobias Fischer,[1,#] Lisa Köhler,[2,#] Florian Trunk,[1,#] Qian-Zhao Xu,[2,‡] Kai-Hong Zhao,[3] Valentin Rohr,[2] Jörg Matysik,[2] Wolfgang Gärtner,[2] Josef Wachtveitl,[1] Chen Song,[2,*] Chavdar Slavov[4,*]

[1]Institute of Physical and Theoretical Chemistry, Goethe University, 60438 Frankfurt, Germany

[2]Institute for Analytical Chemistry, University of Leipzig, 04103 Leipzig, Germany

[3] Key State Laboratory of Agriculture Microbiology, Huazhong Agriculture University Wuhan, 430070 Wuhan, China

[4] Department of Chemistry, University of South Florida, Tampa, FL 33620, USA

†These authors contributed equally to this work and share first authorship

*** Correspondence:**
Chen Song
chen.song@uni-leipzig.de
Chavdar Slavov
chslavov@usf.edu

1 Experimental Procedures

Sample preparation. Preparation of the isolated GAF1-only and bidomain GAF1–PHY constructs of All2699 protein are described elsewhere (Xu et al., 2019; Slavov et al., 2020). For MAS NMR spectroscopy, two All2699 holoproteins in vitro assembled with uniformly ^{13}C- and ^{15}N-labeled PCB [u-[^{13}C,^{15}N]-PCB-All2699(GAF1) and u-[^{13}C,^{15}N]-PCB-All2699(GAF1–PHY)] as lyophilized powder were used (Xu et al., 2019). Prior to lyophilization, the holoproteins were dialyzed against dd-H_2O to remove residual saline. The proteins were illuminated in a ~1.4 mm diameter capillary (100 µL Hamilton syringe) with a 625 nm LED (20 mW, Roithner LaserTechnik, Vienna, Austria) for 10 min at room temperature to ensure the highest Pfr occupancy in the Pr/Pfr photoequilibrium mixture. Aliquots of 500 µL were frozen in liquid N_2 and lyophilized under continuous illumination at 625 nm for 16 h at 10 mbar (Christ Alpha 1-2 LDplus equipment, Osterode am Harz, Germany) (Kim et al., 2020). The lyophilized holoproteins [~3.9 mg of All2699(GAF1) and ~5.4 mg All2699(GAF1–PHY)] were transferred into 4 mm zirconia rotors with the Kel-F insert (active sample volume of ~15 µL) and stored at −20 °C for use. No additional illumination was applied during the NMR acquisition. For ultrafast transient absorption spectroscopy, the in vivo assembled holoproteins were prepared in a final buffer containing 50 mM TRIS, 150 mM NaCl, and 5% Glycerol at pH 8.0.

Stationary spectroscopy. Stationary UV/vis absorption spectra were taken on a Specord S600 absorption spectrometer (Analytik Jena). To convert the sample to the P_r and P_{fr} state the sample was illuminated with an appropriate LED, 730 nm and 625 nm respectively (Thorlabs, 1 W), to achieve photoconversion to the desired state.

Quantum yield determination. The extinction coefficient of GAF1-PHY was obtained, starting from the extinction coefficient of the P_r state (Fischer et al., 2020). The pure P_{fr} spectrum was generated by subtracting a scaled P_r spectrum from the spectrum of the photo-stationary state reached after illumination at 625 nm. Then, a scaling factor S was used to remove the P_r contribution. The obtained spectrum was then multiplied by 1+(S/(1- S)) to account for a complete conversion to the P_{fr} state.

Quantum yields (QY) were calculated by monitoring the irradiation induced absorption changes at 715 nm using a V-650 photospectrometer (Jasco) while illuminating at 730 nm using an LED (1W, Thorlabs) with appropriate filters. The experiment was performed in a 10 mm x 10 mm cuvette filled with 1.3 mL sample volume at an OD of 0.47 at 730 nm at a constant temperature of 20 °C while stirring. To illuminate the sample, the LED light was focused into an optical fiber, emerging directly above the cuvette. The excitation intensity was measured using a P-9719 calibrated light detector (Gigaherz-Optik). The absorption changes were measured every 2 s for 200. For the calculations, only the linear part of the absorption change was used, as photoproduct absorption and sample absorption changes are negligible during this time.

The pH-dependent quantum yields Φ were calculated according to the following equation (1) as described previously (Slavov et al., 2015a).

$$\Phi = \frac{V * h * c * N_A * \Delta A}{P * (1 - 10^{-A_0}) * l * \varepsilon(\lambda_{det}) * t * \lambda_{exc}}$$

Here, V is the volume of the sample, h the Planck constant, c the speed of light, NA the Avogadro constant, ΔA/t the time dependent absorption change, P the light intensity entering the sample cuvette with 8.5 µW, A0 the initial absorption at the wavelength of illumination λ_{exc}, l the optical pathway of the probing light and $\varepsilon(\lambda_{det})$ the molar extinction coefficient of 60012 L mol cm^{-1} at the detection wavelength. The time-dependent absorption changes ΔA/t of 0.00251 OD s^{-1} were obtained from the linear fit (Fig. S1), resulting in an overall QY for the P_{fr} to P_r conversion of GAF1-PHY of 15.8 ± 0.2 %. The experiments were conducted at pH 8, where an additional ms-timescale shunt pathway emerges in GAF1-only, reducing the overall photoconversion QY to 8%, compared to 10% at pH 7, where no shunt channel is observed (Trunk et al., 2025). However, since this loss channel operates on a slower timescale and does not affect the primary photochemical reaction, we used the 10% QY from the pH 7 conditions as the limit in the kinetic modelling. For GAF1-PHY, no indication of a shunt pathways was found, and thus the full 16% QY was used directly for the kinetic modelling (Fischer et al., 2022; Trunk et al., 2025).

NMR spectroscopy. Lyophilized powder samples, where the possible static protein and chromophore conformations are 'frozen out' (Fasshuber et al., 2015), can provide valuable insight into the role of heterogeneity in phytochromes. Despite the partial removal of the hydration shell and the resulting dense packing due to increased hydrophobic protein-protein interactions (Chang and Pikal, 2009), the structural perturbations in small proteins are subtle (Huang et al., 1984; Kim et al., 2020; Köhler et al., 2022), as we showed for GAF1-only and GAF1-PHY Pr where solely minor ^{1}H, ^{13}C and ^{15}N chemical shift differences of the isotopically labelled chromophore occur compared to a frozen solution preparation (Xu et al., 2019; Kim et al., 2020; Köhler et al., 2022).

All solid-state NMR experiments were acquired on a wide-bore Bruker AVANCE-III 400 MHz spectrometer equipped with a 4 mm double-resonance MAS probe (Rheinstetten, Germany). The rotor was inserted into the precooled MAS stator and maintained at an experimental temperature of −23 ± 0.2 °C by a Bruker BVT temperature control unit. The spinning frequency of 8000 ± 2 Hz was applied and regulated by a pneumatic controller. Optimized 1H and 13C π/2 pulse lengths were 2.5 and 3.1 µs, respectively. ^{13}C transverse magnetization created by ramped cross-polarization (CP, 100–70%) was transferred from 1H with an optimal contact time of 2 ms. A r.f. lock field of 64.7 kHz was applied on ^{13}C, fulfilling the Hartmann-Hahn condition. The 2D ^{13}C dipolar-assisted rotational resonance (DARR) spectra were recorded with 114 increments, accumulating 1424 scans in each indirect slice, a relaxation delay of 2.2 s and an optimized mixing time of 50 ms. ^{13}C–^{1}H dipolar interaction was recovered by continuous wave irradiation at r.f. field 16.7 kHz, satisfying the n = +2 rotary-resonance condition. A swept-frequency two-pulse phase modulation heteronuclear decoupling (Thakur et al., 2006) at a ^{1}H r.f. field of 95.6 kHz was used during the acquisition. A 45°-shifted squared sine-bell window function and zero-filling to 1024 points was applied in the indirect dimension prior to Fourier transformation. In the direct dimension, a 90°-shifted squared sine-bell function and zero-filling to 4096 points was applied. All spectra were externally referenced to the COO− signal of solid L-tyrosine·HCl at 172.1 ppm. The data was preprocessed with Bruker Topspin 4.0.1 and further analyzed with MestReNova 14.1.0 (Mestrelab Research, Santiago de Compostella, Spain).

Both P_{fr} DARR spectra were obtained by subtraction of the corresponding P_r spectra from those of the P_r/P_{fr} (0.45:0.55) photoequilibrium mixture used in this study with an appropriate weighting constant of ~0.45. Specifically, the raw data of the P_r dataset were subtracted from those of the P_r/P_{fr} mixture using the standard 'Add / subtract' module embedded in the Topspin program. The 2D P_r and P_r/P_{fr} datasets have equal sizes. The multiplication factor (ALPHA) of the 2D spectrum of P_r/P_{fr} mixture was set to be 1, and the multiplication factor (GAMMA) of the P_r spectrum was tuned down from −0.35 to −0.55. The optimal GAMMA value (P_r weighting constant) was found to be at −0.45 with the complete disappearance of well-resolved P_r-only correlations.

Ultrafast transient absorption spectroscopy. The time-resolved transient absorption experiments were performed using a home-built pump-probe setup which was described previously elsewhere (Slavov et al., 2015a). In the set-up a Ti:Sa amplifier system (Clark, MXR-CPA-iSeries) was used to generate the fundamental laser pulses (775 nm, 130 fs, 1 kHz) which are used to generate the pump and probe pulses. The pump pulses are generated using a home-built two stage NOPA (noncollinear optical parametric amplifier) with a prism compressor in between the two NOPA stages for pulse compression. For probing, white-light continuum pulses were generated by focusing the laser fundamental into a CaF_2-crystal (5 mm). These probe pulses are then split into a probe and reference beam where the probe beam was focused at the sample position, collected and guided into a spectrograph while the reference beam was guided into a second spectrograph directly. The spectrographs (AMKO Multimode) are equipped with 600 grooves/mm gratings blazed at 500 nm and a photodiode array with a detection range of 400-750 nm. A time resolution of ~100 fs for the experiment was estimated from the pump-probe cross correlation. The experiments were conducted under magic angle conditions (54.7° pump-probe polarization difference). The sample was positioned inside the probe beam in a fused silica cuvette with an optical path length of 1 mm and was continuously moved at high speed in two orthogonal directions within a plane perpendicular to the probe beam to avoid accumulation of photoproducts. Both samples were excited at 710 nm using 70 nJ/pulse. The probe energy was 10-20 pJ/nm or lower. The sample was irradiated continuously with a high-power 625 nm LED (Thorlabs, 1 W) to keep it in the P_{fr} state.

The analysis of the transient absorption data was performed using OPTIMUS (http://www.optimus.optimusfit.org/) (Slavov et al., 2015b). Global lifetime analysis is a model dependent method where the transients at all detection wavelengths are analyzed simultaneously using set of exponentials associated with a certain kinetic model. As a model-independent approach, LDA uses a set of 100 exponential functions with set lifetimes which are equally distributed on a log 10 scale to determine the pre-exponential amplitudes. By displaying these amplitudes in a contour plot for all detection wavelengths, a lifetime density map (LDM) (Croce et al., 2001) is obtained which can be read similarly to decay-associated spectra. Negative (blue) amplitudes account for the rise of excited state absorption (ESA), photoproduct absorption (PA) and the decay of ground state bleach (GSB) or stimulated emission (SE). Positive amplitudes correspond to rise of GSB or SE and the decay of excited state or product absorption.

Kinetic models result in species-associated difference spectra (SADS) which contain pure spectral information on the species in the kinetic model and the kinetic rates of the individual reaction steps (van Stokkum et al., 2004; Slavov et al., 2015b). To limit the parameter space, we constrained the quantum yields of formation of the last observable states to those determined as described above. This

aids in determination of an appropriate model. Naturally, kinetic modelling is prone to over-parametrization resulting in non-unique solutions, thus the adequacy of the models was evaluated by examining whether the SADS and kinetic rates are physically reasonable.

2 References

Chang, L. (Lucy), and Pikal, M. J. (2009). Mechanisms of protein stabilization in the solid state. *J. Pharm. Sci.* 98, 2886–2908. doi: 10.1002/jps.21825

Croce, R., Müller, M. G., Bassi, R., and Holzwarth, A. R. (2001). Carotenoid-to-chlorophyll energy transfer in recombinant major light-harvesting complex (LHCII) of higher plants. I. Femtosecond transient absorption measurements. *Biophys. J.* 80, 901–915. doi: 10.1016/S0006-3495(01)76069-9

Fasshuber, H. K., Lakomek, N., Habenstein, B., Loquet, A., Shi, C., Giller, K., et al. (2015). Structural heterogeneity in microcrystalline ubiquitin studied by solid-state NMR. *Protein Sci.* 24, 592–598. doi: 10.1002/pro.2654

Fischer, T., Köhler, L., Ott, T., Song, C., Wachtveitl, J., and Slavov, C. (2022). Influence of the PHY domain on the ms-photoconversion dynamics of a knotless phytochrome. *Photochem. Photobiol. Sci.* 21, 1627–1636. doi: 10.1007/s43630-022-00245-9

Fischer, T., Xu, Q.-Z., Zhao, K.-H., Gärtner, W., Slavov, C., and Wachtveitl, J. (2020). Effect of the PHY Domain on the Photoisomerization Step of the Forward Pr→Pfr Conversion of a Knotless Phytochrome. *Chem. - Eur. J.*, 17261–17266. doi: 10.1002/chem.202003138

Huang, T. H., Bachovchin, W. W., Griffin, R. G., and Dobson, C. M. (1984). High-resolution nitrogen-15 nuclear magnetic resonance studies of .alpha.-lytic protease in solid state. Direct comparison of enzyme structure in solution and solid states. *Biochemistry* 23, 5933–5937. doi: 10.1021/bi00320a007

Kim, Y., Xu, Q.-Z., Zhao, K.-H., Gärtner, W., Matysik, J., and Song, C. (2020). Lyophilization Reveals a Multitude of Structural Conformations in the Chromophore of a Cph2-like Phytochrome. *J. Phys. Chem. B* 124, 7115–7127. doi: 10.1021/acs.jpcb.0c03431

Köhler, L., Gärtner, W., Matysik, J., and Song, C. (2022). Long-Term Preservation of Short-Lived Photoproducts of Phytochromes at Room Temperature. *ChemPhotoChem* 6, e202100220. doi: 10.1002/cptc.202100220

Slavov, C., Bellakbil, N., Wahl, J., Mayer, K., Rück-Braun, K., Burghardt, I., et al. (2015a). Ultrafast coherent oscillations reveal a reactive mode in the ring-opening reaction of fulgides. *Phys. Chem. Chem. Phys.* 17, 14045–14053. doi: 10.1039/C5CP01878A

Slavov, C., Fischer, T., Barnoy, A., Shin, H., Rao, A. G., Wiebeler, C., et al. (2020). The interplay between chromophore and protein determines the extended excited state dynamics in a single-domain phytochrome. *Proc. Natl. Acad. Sci.* 117, 16356–16362. doi: 10.1073/pnas.1921706117

Slavov, C., Hartmann, H., and Wachtveitl, J. (2015b). Implementation and Evaluation of Data Analysis Strategies for Time-Resolved Optical Spectroscopy. *Anal. Chem.* 87, 2328–2336. doi: 10.1021/ac504348h

Thakur, R. S., Kurur, N. D., and Madhu, P. K. (2006). Swept-frequency two-pulse phase modulation for heteronuclear dipolar decoupling in solid-state NMR. *Chem. Phys. Lett.* 426, 459–463. doi: 10.1016/j.cplett.2006.06.007

Trunk, F., Köhler, L., Fischer, T., Gärtner, W., Song, C., Slavov, C., et al. (2025). Single GAF Domain Phytochrome Exhibits a pH-Dependent Shunt on the Millisecond Timescale. *ChemPhysChem* 26, e202401022. doi: 10.1002/cphc.202401022

van Stokkum, I. H. M., Larsen, D. S., and van Grondelle, R. (2004). Global and target analysis of time-resolved spectra. *Biochim. Biophys. Acta BBA - Bioenerg.* 1657, 82–104. doi: 10.1016/j.bbabio.2004.04.011

Xu, Q.-Z., Bielytskyi, P., Otis, J., Lang, C., Hughes, J., Zhao, K.-H., et al. (2019). MAS NMR on a Red/Far-Red Photochromic Cyanobacteriochrome All2699 from Nostoc. *Int. J. Mol. Sci.* 20, 3656. doi: 10.3390/ijms20153656

3 Supplementary Figures

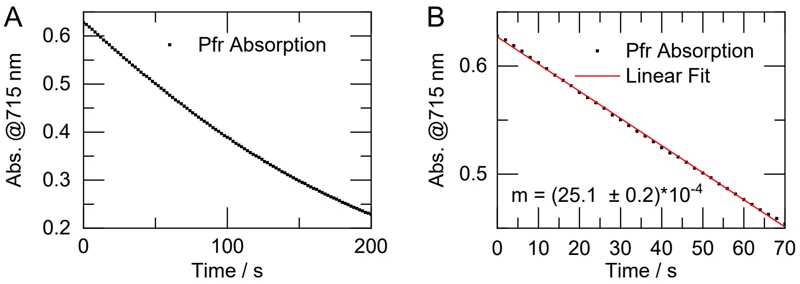

Fig. S1. (a) Time-dependent absorption changes of GAF1-PHY monitored at 715 nm upon illumination at 730 nm. (b) Linear part (from 0 to 70 s) of the data shown in a, including the obtained slope m = ΔA/t and fit error used for quantum yield calculation.

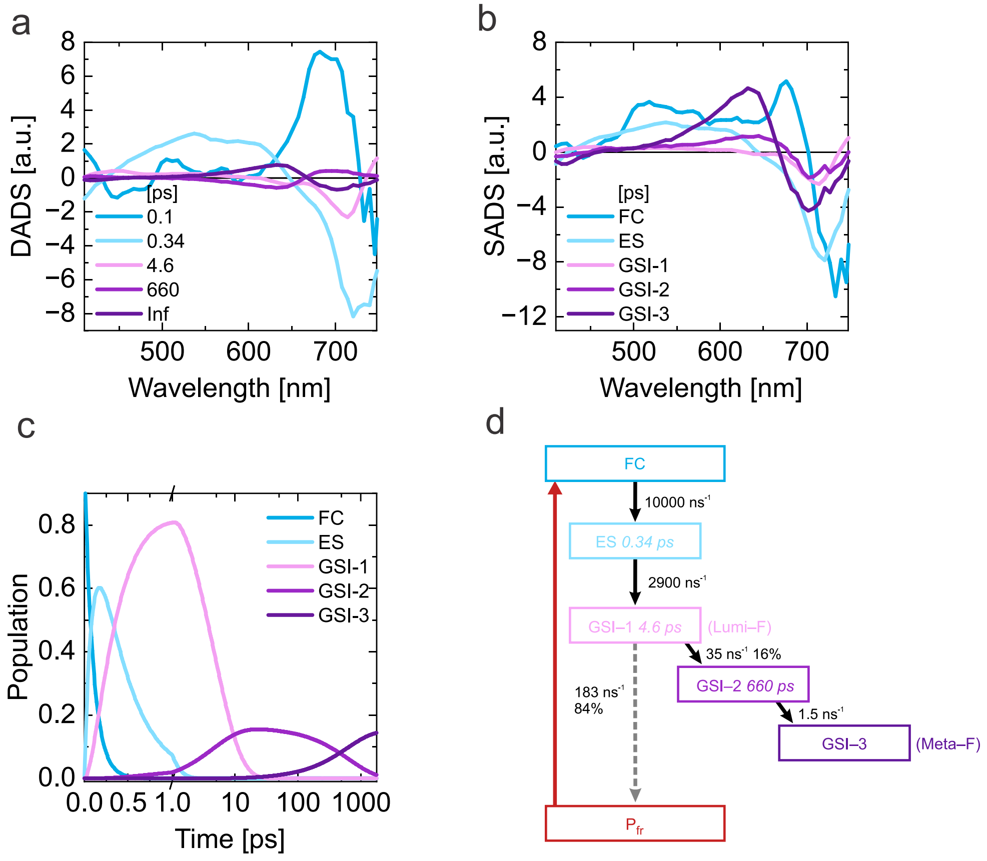

Fig. S2. Kinetic model of GAF1-PHY P_{fr} dynamics. (a) Decay-associated difference spectra. (b) Species-associated difference spectra. (c) Populations throughout the observed time window obtained from the kinetic model. (d) Schematic representation of the best kinetic model to describe the GAF1-PHY P_{fr} dynamics, displaying the obtained rates (in ns^{-1}) and lifetimes (in ps) as well as the branching efficiencies. The model was restricted to an overall QY of 16%. Ground state recovery (dashed line) and branching to Lumi-F occur from GSI-1.

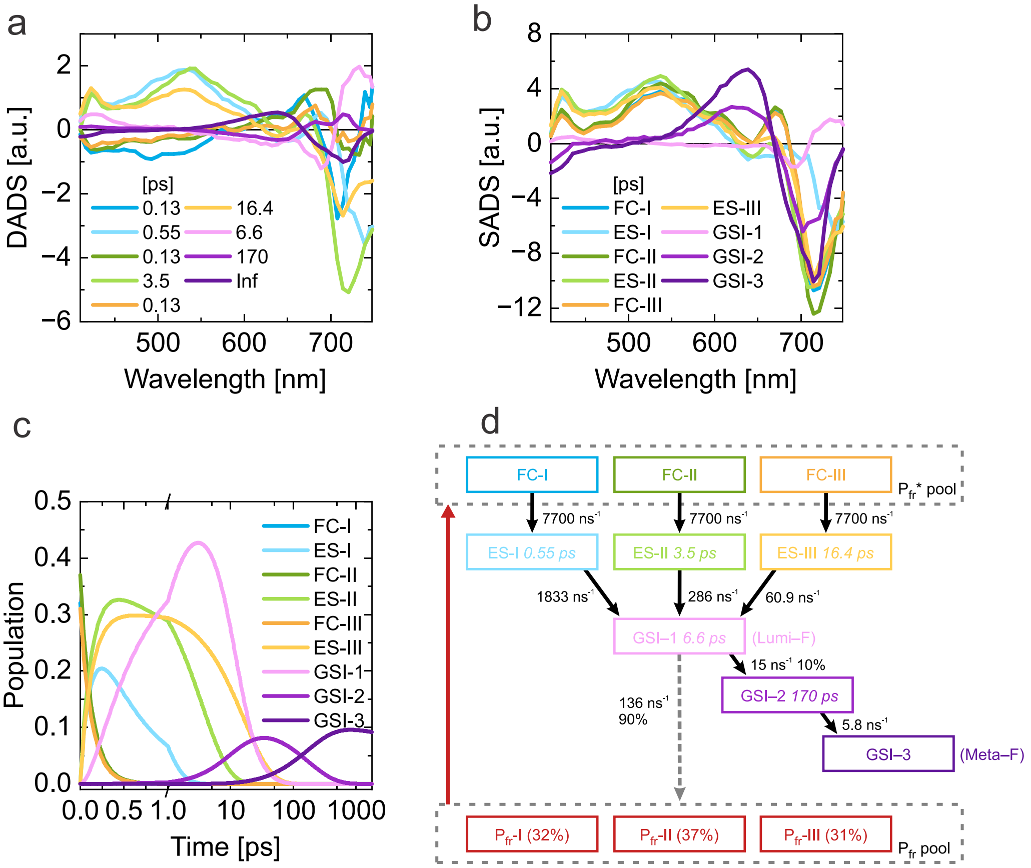

Fig. S3. Kinetic model of GAF1-only P_{fr} dynamics. (a) Decay-associated difference spectra. (b) Species-associated difference spectra. (c) Populations throughout the observed time window obtained from the kinetic model. (d) Schematic representation of the kinetic model to describe the GAF1-only P_{fr} dynamics, displaying the obtained rates (in ns^{-1}) and lifetimes (in ps) as well as branching efficiencies. The model was restricted to an overall QY of 10%. Ground state recovery (dashed arrow) and branching to Lumi-F occur from GSI-1. Given that the FC SADS are spectrally indistinguishable, and we find no evidence for appreciable differences in the absorption cross sections of the three subpopulations, it is reasonable to assume comparable excitation selectivity. Accordingly, the fitted excitation vector (0.32:0.37:0.31 for FC-I:FC-II:FC-III) can be taken as a practical estimate for the ground-state P_{fr}-pool distribution.

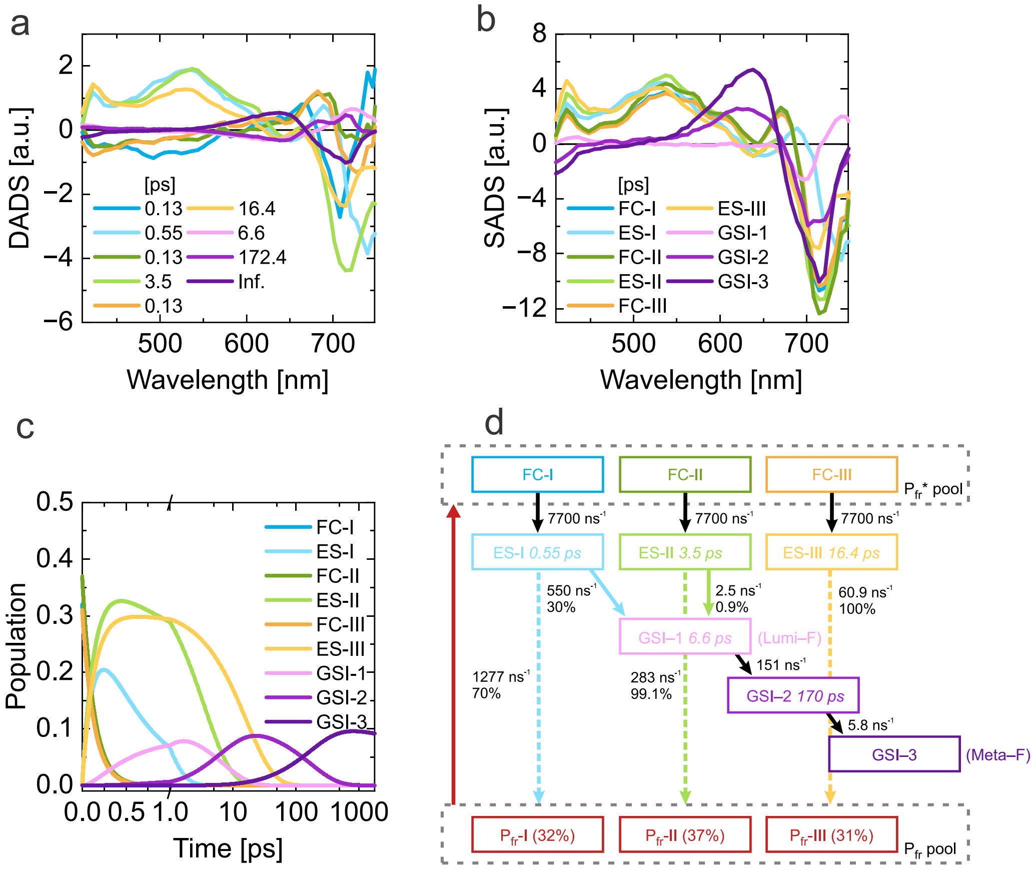

Fig. S4. Alternative kinetic model of GAF1-only P_{fr} dynamics for extracting the quantum yield contribution of each population. (a) Decay-associated difference spectra. (b) Species-associated difference spectra. (c) Populations throughout the observed time window obtained from the kinetic model. (d) Schematic representation of the kinetic model to describe the GAF1-only P_{fr} dynamics, displaying the obtained rates (in ns^{-1}) and lifetimes (in ps) as well as branching efficiencies. The model was restricted to an overall QY of 10%. Ground state recovery channels (dashed lines) were set to occur directly from ES-I-III, yielding population specific product formation efficiencies of ~30% (ES-I), ~0.9% (ES-II), and ~0% (ES-III) with population-weighted total of 10%. In this model, product branching is intentionally implemented to occur at the excited-state level to avoid tripling the GSI-1-GSI-3 states and associated rates for each population, which would overparameterize the model. The excitation vector is defined in the caption of Fig. S3.

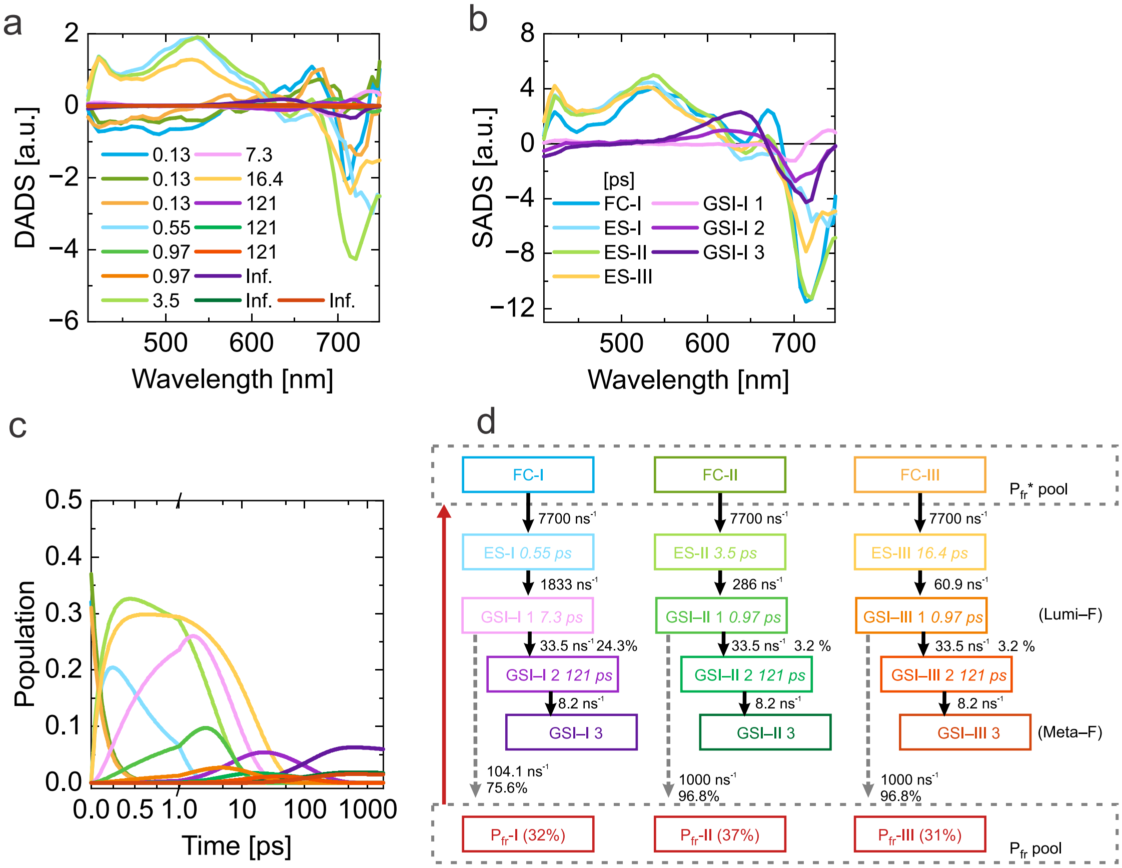

Fig. S5. Alternative fully heterogenous kinetic model of GAF1-only P_{fr} dynamics for extracting the quantum yield contribution of each population. (a) Decay-associated difference spectra. (b) Species-associated difference spectra. (c) Populations throughout the observed time window obtained from the kinetic model. (d) Schematic representation of the kinetic model to describe the GAF1-only P_{fr} dynamics, displaying the obtained rates (in ns^{-1}) and lifetimes (in ps) as well as branching efficiencies. The model was restricted to an overall QY of 10%. To alleviate the limited identifiability arising from the strongly increased number of model parameters, the SADS of corresponding ground-state intermediates were constrained to be identical across populations, and so were the rates along the productive branches toward Meta-F. Ground-state recovery (dashed arrows) was implemented from GSI-I 1-3, yielding apparent efficiencies of ~24.3% (I) and ~3.2% (II and III). Notably, the recovery rates for GSI-II 1 and GSI-III 1 reach the imposed upper bound (1000 ns^{-1}), indicating that these pathways are driven by the constraints rather than uniquely determined by the data. This artificially enhances the efficiencies of populations II and III at the expense of the efficiency of population I, and thus the recovered values cannot be used as reliable estimates. We therefore base the discussion on the more conservative, better-identified estimates from the simplified model in Fig. S4. The excitation vector is defined in Fig. S3.

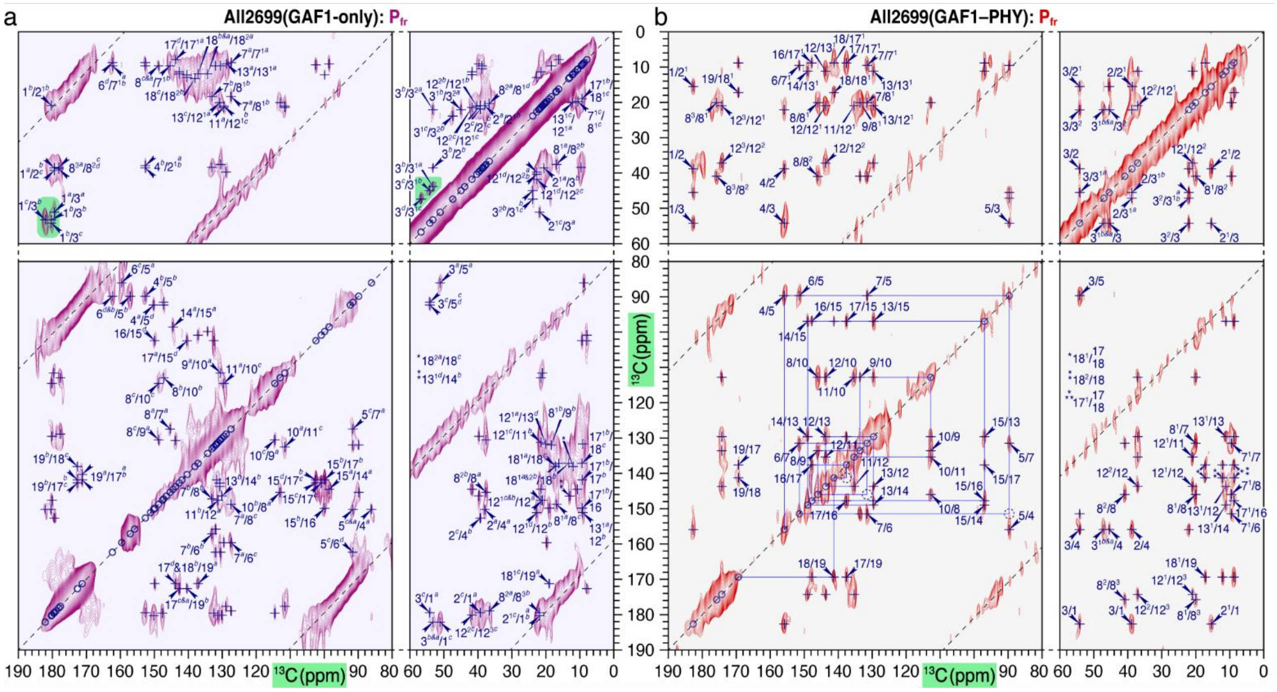

Fig. S6. Structural heterogeneity vs homogeneity at bilin carbons in the two P_{fr} photoproducts of All2699. 2D ^{13}C–^{13}C DARR spectra of (a) GAF1-only and (b) bidomain GAF1–PHY lyophilizates. Calculated spectra of the pure P_{fr} states were obtained by subtracting the corresponding P_r spectra at 100% occupancy (Kim et al., 2020) from those of the P_r/P_{fr} photoequilibrium mixtures used in this study with an appropriate weighting constant of ~0.45. Both short- and long-range carbon pairs of the PCB chromophore (see Fig. 1c for numbering) are assigned with labels in blue. Observed ^{13}C signal splittings are indicated by superscripts a, b, c, and so on from the high- to low-field side. The solid blue lines shown in panel b indicate sequence of short-range (directly-bonded) correlations for tracing ^{13}C connectivities of the π-conjugated C4–C19 system of the bilin in the GAF1–PHY construct.

4 Supplementary Tables

Table S1. ^{13}C chemical shifts of the PCB chromophore of ring *A* and *B* incorporated in GAF1-only and GAF1–PHY) in the respective P_{fr} photoproducts as lyophilized powder (illustrated in Figure 1c). Published ^{13}C data of the two All2699 constructs in the corresponding P_r dark states are listed for reference. The ^{13}C chemical shift changes of the chromophore associated with the presence of the PHY domain ($\Delta\delta^c$) are listed for both P_r dark states and P_{fr} photoproducts and illustrated in Figure 1d.

	PCB carbons	^{13}C chemical shift (ppm)								$\Delta\delta^c$ (ppm)	
		GAF1-only				GAF1-PHY				(GAF1-PHY) − GAF1	
		P_r	Mean value	P_{fr}	Mean value	P_r	Mean value	P_{fr}	Mean value	P_r	P_{fr}
ring A	**1**	182.0 (1^{a})<br>183.8 (1^{b})<br>185.8 (1^{c})	183.9	179.4 (1^{a})<br>180.4 (1^{b})<br>182.1 (1^{c})	180.6	181.6 (1^{a})<br>183.6 (1^{b})<br>-	182.6	182.6	182.6	**−1.3**	**+2.0**
ring A	**2**	36.7 (2^{a})<br>37.2 (2^{b})<br>37.8 (2^{c})<br>38.1 (2^{d})	37.5	37.9 (2^{a})<br>38.6 (2^{b})<br>39.3 (2^{c})<br>-	38.6	37.1 (2^{a})<br>38.8 (2^{b})<br>-<br>-	38.0	38.7	38.7	**+0.5**	**+0.1**
ring A	**2^{1}**	17.0 (2^{1a})<br>18.2 (2^{1b})<br>-	17.6	20.5 (2^{1a})<br>20.9 (2^{1b})<br>21.8 (2^{1c})	21.1	17.0	17.0	15.4	15.4	**−0.6**	**−5.7**
ring A	**3**	51.2 (3^{a})<br>53.1 (3^{b})<br>53.8 (3^{c})<br>54.8 (3^{d})	53.2	51.1 (3^{a})<br>53.2 (3^{b})<br>54.2 (3^{c})<br>56.7 (3^{d})	53.8	51.8 (3^{a})<br>53.5 (3^{b})<br>54.1 (3^{c})<br>-	53.1	54.2	54.2	**−0.1**	**+0.4**
ring A	**3^{1}**	42.6 (3^{1}a)<br>45.4 (3^{1b})<br>47.8 (3^{1c})	45.3	43.8 (3^{1a})<br>44.9 (3^{1b})<br>47.4 (3^{1c})	45.4	43.9 (3^{1a})<br>46.7 (3^{1b})<br>-	45.3	45.4 (3^{1a})<br>47.1 (3^{1b})<br>-	46.3	**0.0**	**+0.9**
ring A	**3^{2}**	21.2 (3^{2a})<br>22.2 (3^{2b})	21.7	22.0 (3^{2a})<br>23.8 (3^{2b})	22.9	22.5	22.5	22.0	22.0	**+0.8**	**−0.9**
ring A	**4**	151.1 (4^{a})<br>153.8 (4^{b})<br>156.6 (4^{c})	153.8	150.2 (4^{a})<br>152.6 (4^{b})<br>-	151.4	155.6	155.6	155.9	155.9	**+1.8**	**+4.5**
**A–B**	**5^{1}**	89.5 (5^{a})<br>90.6 (5^{b})<br>91.2 (5^{c})<br>93.3 (5^{d})	91.7	86.1 (5^{a})<br>89.9 (5^{b})<br>91.6 (5^{c})<br>92.3 (5^{d})	90.0	87.3 (5^{a})<br>88.1 (5^{b})<br>89.5 (5^{c})<br>-	83.3	89.6	89.6	**−3.4**	**−0.4**

		94.1 (5^{c})		-		-					
	**6**	159.1	159.1	155.8 (6^{a})<br>157.1 (6^{b})<br>159.6 (6^{c})<br>162.4 (6^{d})	159.7	150.6 (6^{a})<br>152.0 (6^{b})<br>-<br>-	151.3	151.4	159.1	**−7.8**	**−7.3**
	**7**	132.1 (7^{a})<br>132.6 (7^{b})<br>134.2 (7^{c})	133.0	127.4 (7^{a})<br>133.0 (7^{b})<br>-	130.2	130.7	130.7	131.4	131.4	**−2.3**	**+1.2**
	**7^{1}**	9.7 (7^{1a})<br>10.2 (7^{1b})<br>-	10.0	8.8 (7^{1a})<br>9.6 (7^{1b})<br>10.6 (7^{1c})	182.0	9.2 (7^{1a})<br>10.0 (7^{1b})<br>-	9.6	9.5	9.5	**−0.4**	**−0.2**
	**8**	142.2 (8^{a})<br>144.4 (8^{b})<br>147.5 (8^{c})	144.7	145.4 (8^{a})<br>147.2 (8^{b})<br>148.8 (8^{c})	147.1	145.6 (8^{a})<br>146.4 (8^{b})<br>-	146.0	146.0	146.0	**+1.3**	**−1.1**
**ring B**	**8^{1}**	19.0 (8^{1a})<br>19.5 (8^{1b})<br>21.8 (8^{1c})<br>22.7 (8^{1d})	20.8	16.8 (8^{1a})<br>18.4 (8^{1b})<br>19.7 (8^{1c})<br>20.4 (8^{1d})	18.8	20.2 (8^{1a})<br>21.6 (8^{1b})<br>-<br>-	20.9	20.1	20.1	**+0.1**	**+1.3**
	**8^{2}**	39.2 (8^{2a})<br>40.1 (8^{2b})<br>-<br>-	39.7	36.7 (8^{2a})<br>37.6 (8^{2b})<br>38.3 (8^{2c})<br>38.8 (8^{2d})	37.9	39.2 (8^{2a})<br>40.8 (8^{2b})<br>-<br>-	40.0	40.8	40.8	**+0.3**	**+3.0**
	**8^{3}**	178.6 (8^{3a})<br>179.8 (8^{3b})<br>180.8 (8^{3c})<br>181.4 (8^{3d})	180.2	178.1 (8^{3a})<br>179.0 (8^{3b})<br>-<br>-	178.6	178.3 (8^{3a})<br>180.3 (8^{3b})<br>-<br>-	179.3	175.8	175.8	**−0.8**	**−2.8**
	**9**	127.2 (9^{a})<br>128.4 (9^{b})<br>129.5 (9^{c})<br>130.8 (9^{d})	129.0	130.5 (9^{a})<br>131.9 (9^{b})<br>-<br>-	131.2	128.3 (9^{a})<br>129.1 (9^{b})<br>-<br>-	128.7	133.5	133.5	**−0.3**	**+2.3**

Table S2. ^{13}C chemical shifts of the PCB chromophore of ring *C* and *D* incorporated in GAF1-only and GAF1-PHY in the respective P_{fr} photoproducts as lyophilized powder (illustrated in Figure 1c). Published ^{13}C data of the two All2699 constructs in the corresponding P_r dark states are listed for reference. The ^{13}C chemical shift changes of the chromophore associated with the presence of the PHY domain ($\Delta\delta^c$) are listed for both P_r dark states and P_{fr} photoproducts and illustrated in Figure 1d.

PCB carbons		^{13}C chemical shift (ppm)								$\Delta\delta^c$ (ppm)	
		GAF1-only				GAF1-PHY				(GAF1-PHY) − GAF1	
		P_r	Mean value	P_{fr}	Mean value	P_r	Mean value	P_{fr}	Mean value	P_r	P_{fr}
B-C	**10**	111.2 (10^{a})<br>112.9 (10^{b})<br>114.0 (10^{c})<br>116.1 (10^{d})	113.6	111.6 (10^{a})<br>112.9 (10^{b})<br>114.5 (10^{c})<br>-	113.0	113.1 (10^{a})<br>113.9 (10^{b})<br>-<br>-	113.5	112.8	112.8	**−0.1**	**−0.2**
ring C	**11**	130.1 (11^{a})<br>130.7 (11^{b})<br>-	130.4	129.5 (11^{a})<br>131.3 (11^{b})<br>132.5 (11^{c})	131.1	130.3	130.3	135.3	135.3	**−0.1**	**+4.2**
	**12**	145.8 (12^{a})<br>146.8 (12^{b})	146.3	147.6 (12^{a})<br>151.1 (12^{b})	149.4	143.3	143.3	143.7	143.7	**−3.0**	**−5.7**
	**12^1**	20.0 (12^{1a})<br>20.5 (12^{1b})<br>21.0 (12^{1c})<br>-	20.5	20.1 (12^{1a})<br>21.0 (12^{1b})<br>21.5 (12^{1c})<br>22.7 (12^{1d})	21.3	20.0 (12^{1a})<br>21.3 (12^{1b})<br>-<br>-	20.7	20.9	20.9	**+0.1**	**−0.4**
	**12^2**	37.7 (12^{2a})<br>38.2 (12^{2b})<br>38.7 (12^{2c})	38.2	39.7 (12^{2a})<br>40.3 (12^{2b})<br>41.8 (12^{2c})	40.6	37.0 (12^{2a})<br>37.6 (12^{2b})<br>-	37.3	37.1	37.1	**−0.9**	**−3.5**
	**12^3**	178.6 (12^{3a})<br>180.4 (12^{3b})<br>-	179.5	177.6 (12^{3a})<br>179.6 (12^{3b})<br>180.1 (12^{3c})	179.1	177.9 (12^{3a})<br>179.4 (12^{3b})<br>-	178.7	174.2	174.2	**−0.8**	**−4.9**
	**13**	129.7 (13^{a})<br>131.4 (13^{b})<br>132.0 (13^{c})<br>-	131.0	128.9 (13^{a})<br>130.1 (13^{b})<br>130.8 (13^{c})<br>131.7 (13^{d})	130.4	125.7 (13^{a})<br>126.6 (13^{b})<br>-<br>-	126.2	129.6	129.6	**−4.9**	**−0.8**
	**13^1**	10.8 (13^{1a})<br>11.4 (13^{1b})<br>-<br>-	11.1	9.1 (13^{1a})<br>11.4 (13^{1b})<br>12.2 (13^{1c})<br>12.9 (13^{1d})	11.4	9.8	9.8	11.1	11.1	**−1.3**	**−0.3**
	**14**[1]	145.1	145.1	144.5 (14^{a})	144.9	144.9	144.9	149.0	149.0	**−0.2**	**+4.1**

				146.4 (14^{b})							
C-D	**15**	95.2 (15^{a})<br>97.2 (15^{b})<br>97.6 (15^{c})<br>-	96.7	98.6 (15^{a})<br>99.8 (15^{b})<br>100.8 (15^{c})<br>102.4 (15^{d})	100.4	94.1 (15^{a})<br>95.8 (15^{b})<br>-<br>-	95.0	97.0	97.0	**−1.7**	**−3.4**
	**16**	146.9	146.9	149.8	149.8	145.0	145.0	147.8	147.8	**−1.9**	**−2.0**
	**17**	140.5 (17^{a})<br>141.4 (17^{b})<br>142.1 (17^{c})<br>-	141.3	140.4 (17^{a})<br>141.8 (17^{b})<br>142.7 (17^{c})<br>143.8 (17^{d})	142.2	142.2 (17^{a})<br>142.8 (17^{b})<br>-<br>-	142.5	137.6	137.6	**+1.2**	**−4.6**
	**17^1**	8.7 (17^{1a})<br>9.3 (17^{1b})<br>-	9.0	7.9 (17^{1a})<br>9.4 (17^{1b})<br>10.7 (17^{1c})	9.3	8.9 (17^{1a})<br>9.8 (17^{1b})<br>-	9.4	8.8	8.8	**+0.4**	**−0.5**
ring D	**18**	131.9 (18^{a})<br>133.6 (18^{b})<br>-	132.8	134.3 (18^{a})<br>137.2 (18^{b})<br>138.0 (18^{c})	136.5	132.7 (18^{a})<br>133.6 (18^{b})<br>-	133.2	141.2	141.2	**+0.4**	**+4.7**
	**18^1**	16.4 (18^{1a})<br>17.4 (18^{1b})<br>-	16.9	16.2 (18^{1a})<br>17.1 (18^{1b})<br>19.0 (18^{1c})	17.4	15.5	15.5	17.2	17.2	**−1.4**	**−0.2**
	**18^2**	12.9 (18^{2a})<br>14.5 (18^{2b})	13.7	12.0 (18^{2a})<br>13.2 (18^{2b})	12.6	12.6	12.6	12.1	12.1	**−1.1**	**−0.5**
	**19**	173.8 (19^{a})	174.3	171.2 (19^{a})	171.9	173.4	173.4	169.4	169.4	**−0.9**	**−2.5**
